# Intraoperative Incidental Finding of Meckel’s Diverticulum: A Report of Two Cases

**DOI:** 10.7759/cureus.68387

**Published:** 2024-09-01

**Authors:** Carmen Alhaddad, Antoine S Geagea, Sarah Muheiddine, Antoine Kachi

**Affiliations:** 1 General Surgery, Lebanese University Faculty of Medicine, Beirut, LBN; 2 Gastroenterology and Hepatology, Lebanese Hospital Geitaoui University Medical Center, Beirut, LBN; 3 General Surgery, Lebanese Hospital Geitaoui University Medical Center, Beirut, LBN

**Keywords:** diverticulitis, surgical treatment, incidental, resection, meckel diverticulum

## Abstract

Meckel’s diverticulum (MD), a prevalent congenital gastrointestinal anomaly affecting about 2% of the population, arises from the incomplete closure of the vitelline duct. It encompasses all layers of the small intestine and can lead to various complications like obstruction, hemorrhage, and perforation. When symptomatic, it presents challenges in diagnosis due to the low sensitivity of imaging techniques. Comprehensive understanding and accurate diagnosis are crucial for managing the complications associated with MD and forming the scientific rationale for publishing this case report. We present two cases, one of them being the case of a 73-year-old male who presented for an ileostomy closure procedure. Intra-operatively, a 4.5 cm diverticulum was identified 10 cm from the stomatal opening on the efferent limb. This finding led to segmental resection of the intestines. Later, pathology was compatible with MD, which didn’t contain any malignant cells or heterotopic tissue. The second case was that of a 40-year-old female who presented for severe abdominal pain, abdominal distention, and obstipation for two days. Radiographic imaging was suspicious of a foreign object compatible with fish bone with local inflammation in the small bowel. Laparoscopic exploration showed an inflamed MD with fish bone lodged inside. In front of an incidental MD, the decision to resect is still controversial. Those who are against resection of uncomplicated believe that complications from resecting an uncomplicated MD are higher than the complications that arise if resection is not performed. Those who support resection say that the complications that arise following the resection of a complicated MD are worse than those after resecting an incidental one. Criteria have been put in place to help guide the decision for resection.

## Introduction

Meckel’s diverticulum (MD) arises from an incomplete closure of the vitelline duct, a congenital anomaly. It ranks as the most prevalent gastrointestinal malformation from birth, found in roughly 2% of the population. It constitutes a true diverticulum, encompassing all three layers of the small intestine. The complications associated with MD include obstruction, hemorrhage, perforation, diverticulitis, and intussusception [1].

They can be an incidental finding or present with any of the previously mentioned complications. In the latter case, diagnostic imaging is challenging in confirming the diagnosis, seeing the low sensitivity. Computed tomography (CT) has a low sensitivity to be used. Barium meal is more sensitive but can’t be used in the acute setting. The imaging modality that holds the most sensitivity is the pertechnetate scan, which is mostly useful in the presence of gastric heterotopic mucosa [2].

MD poses a concerning risk for cancer development, notably malignant carcinoid tumors. Additionally, it may harbor heterotopic gastric tissue, contributing to significantly lower gastrointestinal tract bleeding. Among individuals with symptomatic MD, males have a higher occurrence compared to females, with a ratio of 1.9:1. This condition tends to manifest more frequently in younger individuals. Treatment for symptomatic MD typically involves resection [3].

## Case presentation

Case 1

We present the case of a 73-year-old male patient who presented to our facility for ileostomy closure three months after undergoing a total colectomy elsewhere.

He is known to have hypertension, type 2 diabetes mellitus, and chronic kidney disease. He isn’t a smoker and drinks alcohol on occasion.

His past surgical history was relevant for total colectomy three months ago and an open cholecystectomy eight weeks ago following an episode of cholecystitis and choledocholithiasis.

His colectomy was initially started laparoscopically and was later converted to an open procedure. His pathology results came back positive for moderately differentiated adenocarcinoma, negative margins, and negative lymph nodes.

As pre-operative preparation, the patient underwent a barium enema through the ileostomy to check the patency of the efferent limb, and a digital rectal exam was performed to rule out any strictures. The barium images showed no outpouching of the observed part of the digestive tract. The pre-operative CT scan showed no abnormalities in the gastrointestinal tract.

**Figure 1:**
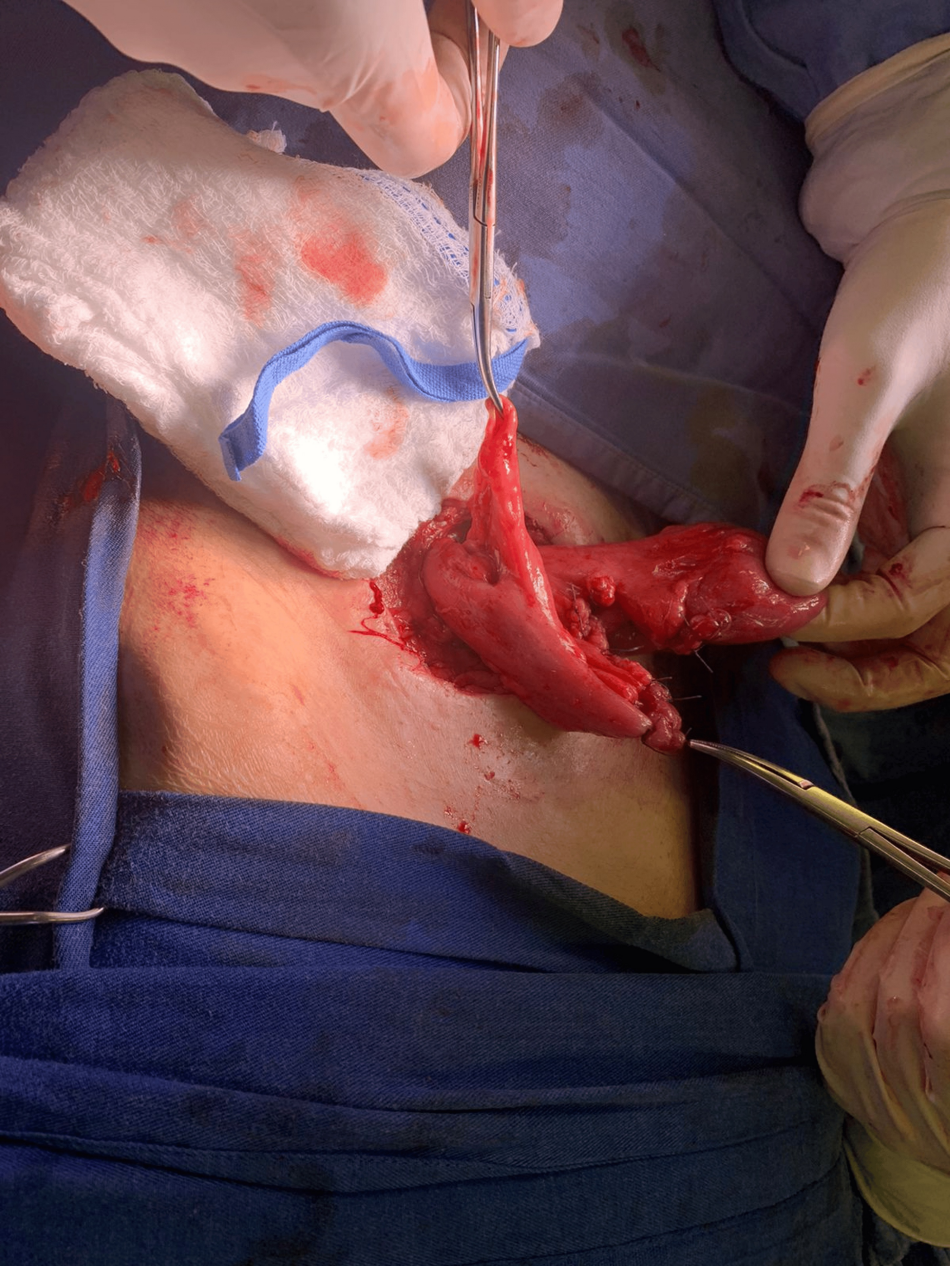
Intraoperative photograph showing a diverticulum 10 cm away from the ileostomy on the efferent limb

During surgery, following suturing of the skin around the ileostomy and elliptical incision around that portion, dissection of the fascia, and liberation of the ileostomy, a diverticulum (Figure 1) was identified 10 cm from the efferent limb. More dissection was performed to allow segmental resection of the diverticulum.

In the post-operative period, the patient had a smooth hospital recovery and was then discharged home.

Pathology reports noted a 4.5 cm long and 1.5 cm in diameter diverticulum whose wall contains all layers of the digestive tract, and the mucosa has inflammatory changes. The results are compatible with MD.

Case 2

**Figure 2:**
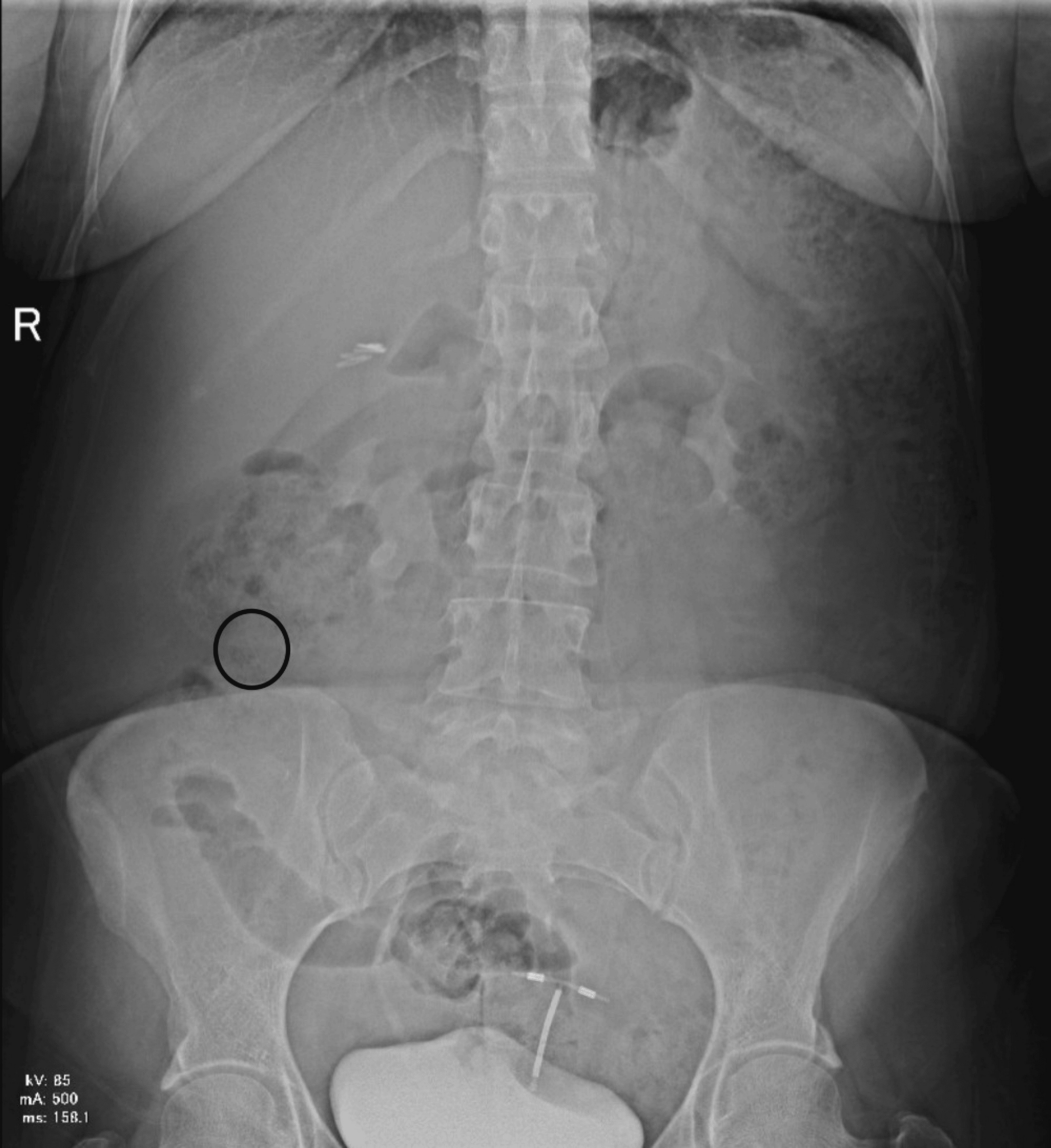
Kidney-ureter-bladder (KUB) image showing the suspicious foreign body in the right flank area (circled in black)

**Figure 3:**
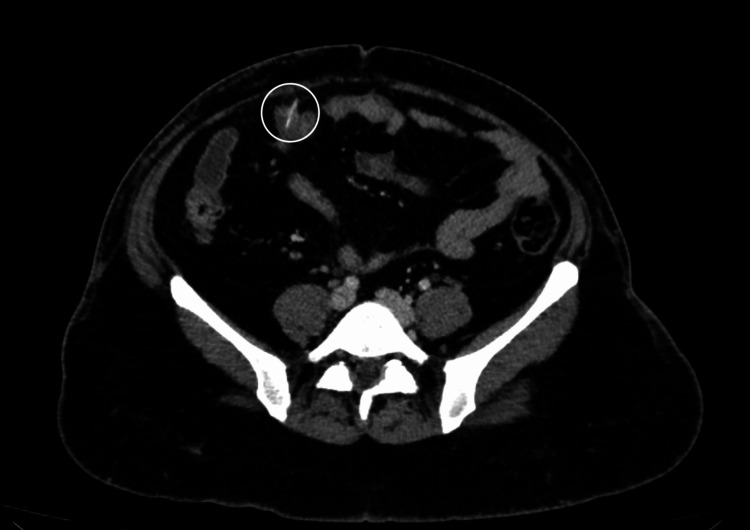
Foreign body identified on CT scan, in the ileal loops, with surrounding area of inflammation (white circle)

**Figure 4:**
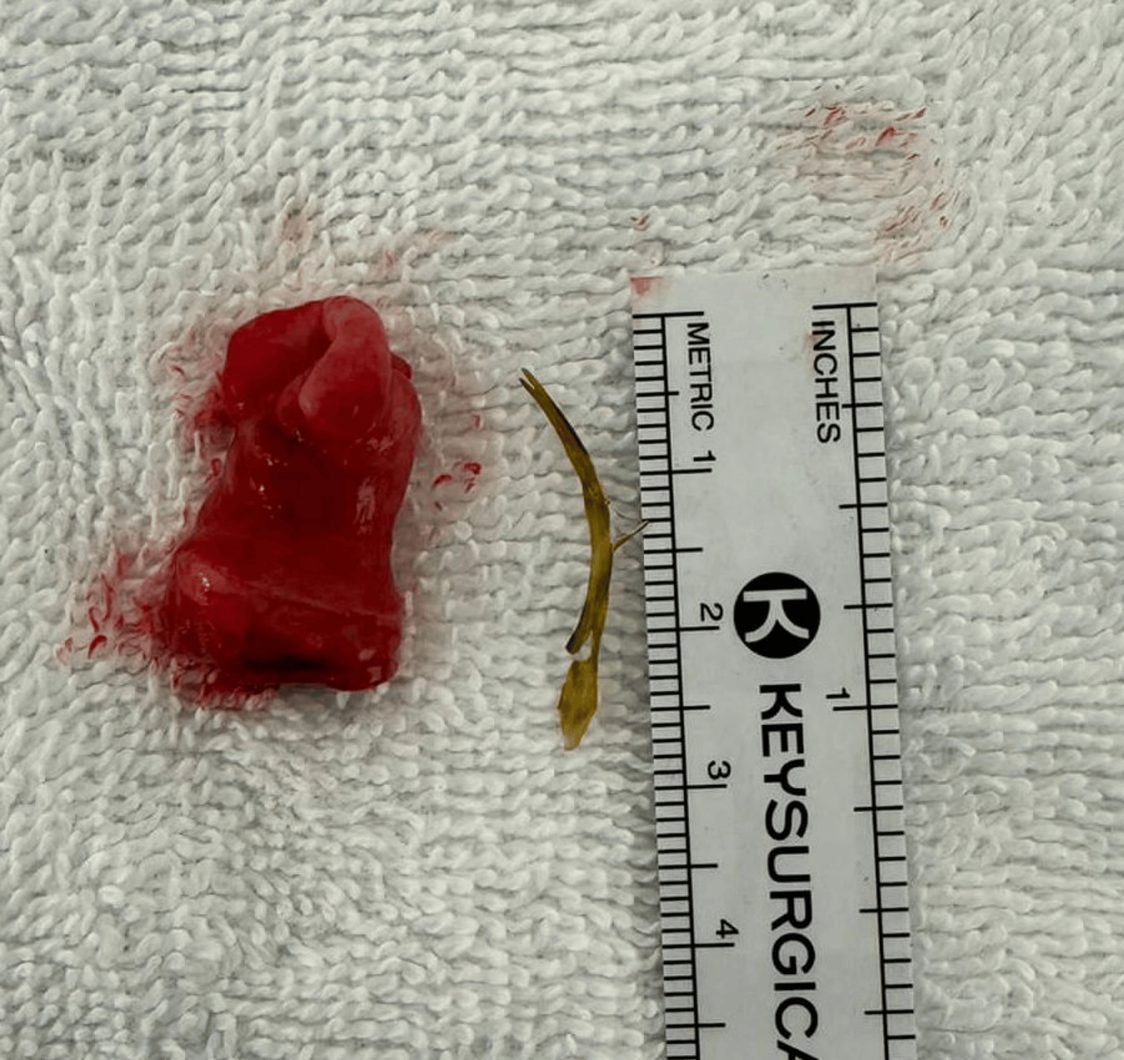
Image showing the resected outpouching (left side) with the extracted foreign body (right side, a fishbone)

The second case is that of a 40-year-old female patient with no relevant previous health history who presented to the Lebanese Hospital Geitaoui’s Emergency Department for abdominal pain of two days origin. The patient described the pain as being diffuse. She had been obstipated for a day and suffered from nausea. On the physical exam, we had a distended abdomen that was diffusely tender. A KUB (Figure 2) was taken and showed a suspicious, elongated, and thin foreign body at the right lower quadrant. Upon further interrogation, the patient revealed that two days ago, she hurriedly ingested fish without properly chewing. A CT scan of the abdomen and pelvic area (Figure 3) showed a needle-like body in the ileum, with signs of local inflammation. Her labs were relevant for elevated white count (14,700) and slightly disturbed liver function tests (LFTs). The patient was then prepared for laparoscopic exploration, segmental resection, and anastomosis. After abdomen insufflation and trocar insertion, a diverticular-like segment was observed during exploration, which showed inflammatory changes. On manipulation, a needle-like structure was extracted (Figure 4), which turned out to be a fishbone. Excision of the diverticular pouch was done with primary closure of the bowel. The patient had a smooth recovery and was discharged home. The pathology report was compatible with an MD with inflammatory changes without malignant changes.

## Discussion

Our cases depict the intraoperative incidental finding of an MD in an asymptomatic patient who had recently undergone an open total colectomy and that of a patient who was suffering from diverticulitis due to a foreign body. The literature is divided when it comes to the decision of an incidental asymptomatic MD resection. Cullen et al. initially supported the resection of incidentally found MD in order to avoid a potential cause of complications later on in life [4]. Zani et al. proved that the post-operative complications of those who underwent incidental MD resection (5.3%) were higher than those who didn’t (1.3%), thus supporting the non-resection of incidental MD [5]. Park et al. established four criteria that support resection the more they are present, and they include patient age (younger than 50 years old), diverticulum length (more than 2 cm), male sex, and abnormal diverticular features [6].

Surgical resection of an MD includes diverticulectomy or segmental resection. The latter is usually preferred in order to avoid leaving residual heterotopic mucosa [7].

In summary, the management of asymptomatic MD remains contentious, with varying opinions on the necessity of prophylactic resection for asymptomatic cases. The approach may differ based on age, risk factors, and clinical considerations, emphasizing the need for personalized decision-making.

## Conclusions

In summary, MD is a condition that affects a minority of the population, and its clinical manifestations are generally non-specific. We report the case of an incidental MD during ileostomy closure in a patient who had undergone open colectomy. The management of incidental asymptomatic MD lacks a clear consensus, and various studies over the years have aimed to establish criteria for deciding whether to resect or retain an asymptomatic MD. In the absence of definitive guidelines, the surgeon relies on expertise and common sense when approaching such cases.
